# Use of Energy Efficient Sensor Networks to Enhance Dynamic Data Gathering Systems: A Comparative Study between Bluetooth and ZigBee

**DOI:** 10.3390/s18061801

**Published:** 2018-06-03

**Authors:** Razvan Andrei Gheorghiu, Valentin Iordache

**Affiliations:** Transports Faculty, Politehnica University of Bucharest, Bucharest 060042, Romania; valentin.iordache@upb.ro

**Keywords:** sensor networks, vehicular communications, ZigBee, Bluetooth, Wi-Fi, data acquisition system, sensors

## Abstract

As road traffic conditions worsen due to the constantly increasing number of cars, traffic management systems are struggling to provide a suitable environment, by gathering all the relevant information from the road network. However, in most cases these are obtained via traffic detectors placed near road junctions, thus providing no information on the conditions in between. A large-scale sensor network using detectors on the majority of vehicles would certainly be capable of providing useful data, but has two major impediments: the equipment installed on the vehicles should be cheap enough (assuming the willingness of private car owners to be a part of the network) and be capable of transferring the required amount of data in due time, as the vehicle passes by the road side unit that acts as interface with the traffic management system. These restrictions reduce the number of technologies that can be used. In this article a series of comprehensive tests have been performed to evaluate the Bluetooth and ZigBee protocols for this purpose from many points of view: handshake time, static and dynamic data transfer (in laboratory conditions and in real traffic conditions). An assessment of the environmental conditions (during tests and probable to be encountered in real conditions) was also provided.

## 1. Introduction

The Internet of Things is a modern concept that allows the connection to the Internet and, hence, remote access and control for many devices that were initially meant to be used locally. This concept, that basically represents the possibility of having a network of different types of devices that are able to work together in an integrated system, may be transferred to the road transport field, supporting the development of new applications, such as dynamic information gathering, or even an integrated concept of Internet of Vehicles.

Communication is the key element in the progress of future applications. Even if it is about the data exchange between a vehicle and a road side unit (RSU) with the purpose of informing the driver about the conditions on the road ahead, the data collected by the vehicle along the path that is downloaded into the RSU, or even collaborative vehicle driving, the communication system is the backbone of each development.

However, communication in the vehicular environment presents many challenges: signal propagation issues, environmental conditions, Doppler shifts depending on the speed of the receiver relative to the transmitter (or vice versa), on how crowded the communication channels are and not least, all the possible interferences. Each of these represent an issue that may have a negative influence on the communication.

There are many studies that have tried to develop a new communication system that will address all (or at least most of) these issues using dedicated communication technologies, such as Dedicated Short Range Communication (DSRC). A useful data collection system in a road environment should be able to collect information from many sources via a multitude of sensors, thus being similar to a large sensor network. The main disadvantage of special technologies is the final cost of the system, being based on expensive solutions, especially for large-scale networks. In addition, the operating cost of the system is relatively high, as the main concern is the signal propagation aspect, and not the energy efficiency. The advantages are not able, in many situations, to offset the disadvantages.

The approach in this paper starts from the disadvantages presented above, thus, in the development of a large sensor network able to collect meaningful data to be used in traffic management, the focus should be on the costs, but having also in mind the communication reliability. If it is expected to extend such system beyond the usage of the public vehicles (that can be included in the system more easily) to private cars, the solution must be inexpensive and provide enough facilities to drivers to convince them to be a part of the data collection network.

The foreseen benefits will be for all the parties involved, as the general traffic management system may obtain more information from the whole network (as opposite of having data collection equipment only in fixed points, like road intersections), but also the drivers may acquire useful information for their trip: incident/accident locations, congested areas, travel speeds, alternative routes, road works, etc.

Modern vehicles already have many installed sensors: rain, light, speed, distance to objects in front or in the rear, etc. Therefore, the components that can acquire information are, in many cases, already installed. The key element is the communication system that must be installed to allow data exchange. The main features on the system, from a private vehicle owner point of view, are purchase costs, operation costs, and benefits obtained.

In this article, the focus will not be on the applications that may be implemented, or the types of sensors that may be used, as there are many studies for these (like [1,2]), but the technology that may be used (and in what conditions will it function) to support such systems.

In Section 2 are presented some brief details about the technologies that were considered. The authors have performed a literature review, trying to find similar analysis for Bluetooth, ZigBee, or even a comparison of the two technologies. In the end is emphasized the main topic of this paper and the original contributions. Section 3 presents all the details about the tests performed: environmental assessment, handshake time, static data transfers and data exchange in motion. In Section 4 the conclusions of these studies are drawn.

## 2. Background and Related Work

There are several wireless technologies that can be used to transmit data from vehicles, with reduced costs, all of them working in the Industrial, Scientific and Medical (ISM) frequency band. Among them we can mention:

- Wi-Fi is the most common wireless communication. Its main disadvantage is the considerable number of users (transmission crowdedness) and interference with other communications. This communication is used in almost all office buildings, but it can be activated on the smartphones inside vehicles. In addition, in some modern vehicles Wi-Fi access is provided, multiplying the devices of this kind that can be detected along the road.
- Bluetooth: is the most common connection method between two portable devices. It is usually found in vehicles, connecting the phone with the audio system, but it’s also used for headset connections. Therefore, a significant number of Bluetooth communications are likely to be found near the road network. Bluetooth’s advantage is the usage of frequency hopping, that continuously search for free channels to be used in data exchange. This ensures the successful data exchange, but the communication time depends heavily on the frequency band congestion.
- ZigBee: is an uncommon technology to be used for vehicle communications, as it was designed for smart home device networks, being capable of fast data transfers (at low data rates) between a substantial number of devices. ZigBee may use fixed (pre-selected) channels, that may be chosen far enough from usual interferences in 2.4 GHz frequency band. ZigBee uses an interference mitigation technique (frequency agility mechanism [3]), that can be divided into three phases: interference detection, channel evaluation and interference mitigation [4]. The main feature of this technology is its very efficient use of energy [3]. As we will present in the following sections, it is proven to be a very reliable communication, more reliable in fact than Wi-Fi or Bluetooth. Adding the layered network topology and the reduced price of the equipment, we consider it to be the most suitable technology to be used in wireless sensor networks.

Considering all these aspects, a comparison between solutions needs to be performed. Although there are several papers dedicated to Bluetooth analysis (such as [5,6,7]), or ZigBee evaluation (such as [8,9]), very few compare these technologies (one probable cause may be that they were developed in the beginning for different purposes). ZigBee is an unusual choice for a vehicular communication protocol, or even communication between moving devices, but has great advantages from the power consumption point of view. In addition, none of the studies found that focused on Bluetooth vs. ZigBee that seemed to be representative for the purposes of this article [10,11,12,13], included comparative evaluation of the communication aspects involving these two technologies, focusing instead on power consumption or just presenting technical specifications from datasheets. Therefore, an in-depth analysis of all the aspects related to communication between two devices appeared as a requirement for this study.

This is the main topic of this paper and the main contribution of the authors to this field. Real-life tests have been performed (not just data taken from datasheets) and the tests made in similar conditions create a complete and real image of the two technologies and possibility to implement them in real traffic applications.

There are several types of influences for these technologies, being based on radio signals, but the most influence on the communication are the limitations of the technology used (in terms of distance, data rate, etc.), and interference with other communications in the same frequency band. As the most known (and, therefore, used) solutions are Wi-Fi, Bluetooth, and ZigBee, is seems the analysis should study the influence of each technology on the others. However, previous work [14,15] has revealed minimum influence on Bluetooth over ZigBee and vice-versa, therefore the tests performed had the goal to evaluate these technologies in a Wi-Fi environment.

## 3. Assessment Tests Performed and Results

For the evaluation of possible usage of Bluetooth and ZigBee technologies, we have tested the handshake time (device connection time) and data transfer time for specific amounts of data. Both are important in a dynamic environment, in which vehicles are moving at high speeds and, therefore the devices must be able to connect and transmit the required information very fast. The tests were performed both in a laboratory environment and on a road network.

### 3.1. Hardware Used

Test equipment is based on Arduino Uno v3 development boards, that allow communication with and control of Bluetooth and ZigBee communication modules which were chosen as following due to their low price and high availability on the market.

**Figure 1:**
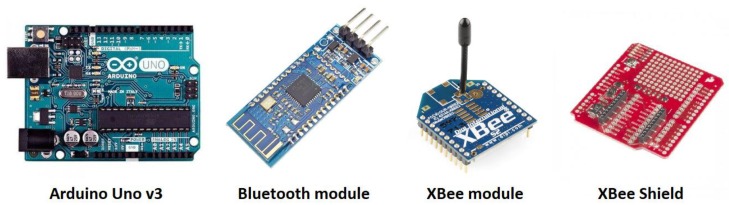
Hardware components.

Bluetooth version 4.0 modules HM-10 with a CC2541 chipset were used (Figure 1), having the following relevant specifications [16,17,18,19]:

- Data rate: up to 2 Mbps.
- RF power: up to 6 dBm (5 mW).
- Electric current consumption: in active mode—8.5 mA, and in sleep mode—400 μA~1.5 mA.
- Power supply: 3.3 V DC, 50 mA.
- Wireless range: up to 100 m.

According to the Bluetooth network specifications, one of the modules was set as Master and the other one as Slave, with search for a pair and connection establishment being set to automatically.

It is to be underlined that Bluetooth v4 was used in these tests due to the fact that it was the state of the art Bluetooth technology available at the beginning of the research project. Although during the meantime the Bluetooth v5.0 specifications were made available for the market, test modules were not available until recently. Some aspects regarding Bluetooth v5 were tested in [20], such as throughput against distance or influence of obstacles, or in [21] for RSSI against distance. However, it must be mentioned that the tests performed revealed only static behaviour (but not in Wi-Fi interference conditions), and future assessments have to be performed to evaluate the improvements of Bluetooth v5 against older versions in sensor networks, using similar conditions to the ones defined in this paper.

For the ZigBee technology, XBee Series 2 with Wire Antenna modules were used (Figure 1), having the following relevant specifications [3,22]:

- Data rate: up to 250 kbps.
- RF power: up to 3 dBm (2 mW).
- Electric current consumption: in active mode—40/45 mA, and in sleep mode—1 μA.
- Power supply: 3.3 V DC.
- Wireless range: up to 120 m.

According to the ZigBee network specifications, one of the modules was set as Coordinator and the other one as End-Device. An XBee Shield was used as an interface between the module and the Arduino board (Figure 1).

To create interference in the 2.4 GHz spectrum on specific channels the authors inserted between the test modules a Wi-Fi router and a computer, and transferred large files with speeds up to 70 Mbps, the maximum data rate that could be achieved with the equipment used. The router was manually set to the desired channel and bandwidth.

### 3.2. Wireless Channels Assessment

**Figure 2:**
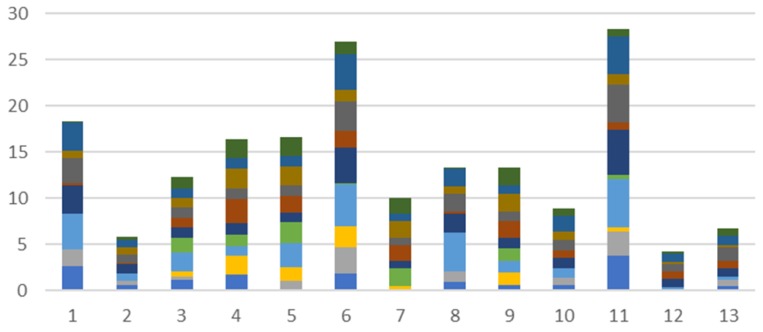
Wi-Fi channel density [24].

In this section, all wireless technologies used are analyzed in terms of interference between each other to determine which channels will be used in the tests. To test the hypothesis about the congested Wi-Fi environment we have performed several tests in the Bucharest road network (Bucharest being the capital city of Romania). The city is ranked in the 5th position among the most crowded cities in the world top according to Tom-Tom [23], making it one of the best places to test field communications in a crowded environment (at least, considering all the communication issues mentioned in Section 2). In this environment, several junctions and public transport routes have been selected, based on the probability for other communications to be present (Wi-Fi, Bluetooth or ZigBee). The test sites were chosen near blocks of flats, or office buildings to maximize the data that could be collected. The aim of this test was to evaluate the communication density and to assess the signal power for each Wi-Fi channel. The results presented in Figure 2 and published in [24] were meant to set the reference for the following interference tests between Wi-Fi and other communication technologies.

**Figure 3:**
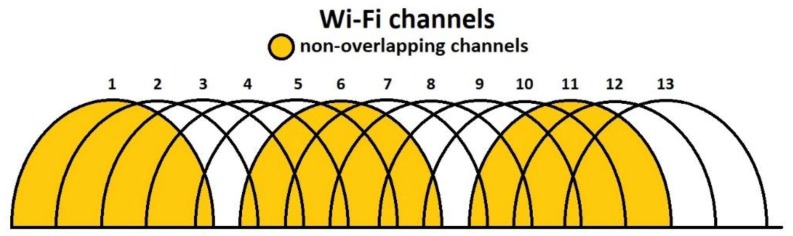
Wi-Fi channel allocation.

From the tests performed on the road network, both for junctions and public transport routes, it resulted that, overall, the most used Wi-Fi channels are 1, 6, and 11 which is a common practice due to the fact they are not overlapping (as detailed in [25,26] and seen in Figure 3) and interference from nearby devices can be avoided, but as the number of devices is continuously increasing, and routers implement modern algorithms to switch from one channel to another in case of congested communications, so changes may appear in this pattern and Wi-Fi communications tend to exceed the typical spectrum usage and extend to other, less congested, frequencies, although they are overlapping each other.

**Figure 4:**
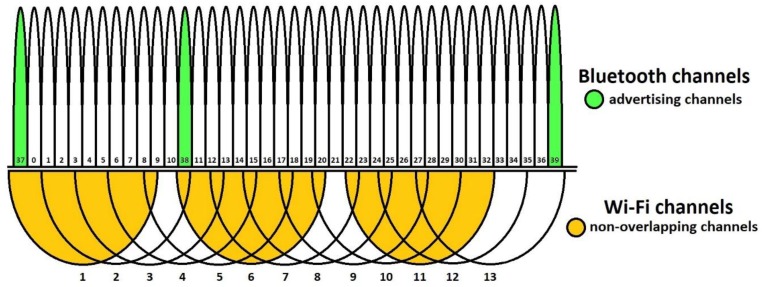
Bluetooth vs. Wi-Fi channel allocation.

The main influence of this Wi-Fi spectrum is on Bluetooth technology, as it uses three advertising channels and two of them are overlapping Wi-Fi channels 1 and 6 (as detailed in [27] and seen in Figure 4). 

The great advantage of this technology is that it uses Adaptive Frequency Hopping, a technique that automatically selects the communication channel according to the detected degree of interference.

**Figure 5:**
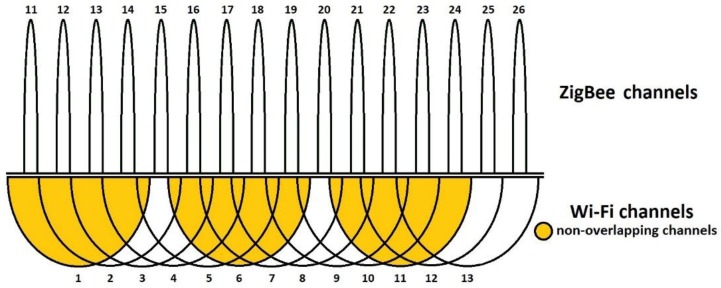
ZigBee vs. Wi-Fi channel allocation.

ZigBee, as opposed to Bluetooth, may communicate on a specific channel, making it (at least in theory) sensitive to other communications in the same frequency band. For the evaluation of this interference, we considered the ZigBee channels 12, 17 and 22, which are closest to the center frequency of the Wi-Fi channels 1, 6 and 11, respectively, and clearly overlap with them (as detailed in [28] and seen in Figure 5). In addition to these, we have tested ZigBee channels 25 (0X19) and 26 (0x1A), to verify that they have the least probability of being influenced by any of the selected Wi-Fi channels.

### 3.3. Handshake Time Tests

The first tests that have been performed for the evaluation of each technology employed measuring the handshake time. Connection time evaluation was performed under laboratory conditions, inside a building, in an open space, with direct line of sight between the emitter and receiver. This allowed the monitoring and control of the radio environment, especially the communication that provide potential interferences.

**Figure 6:**
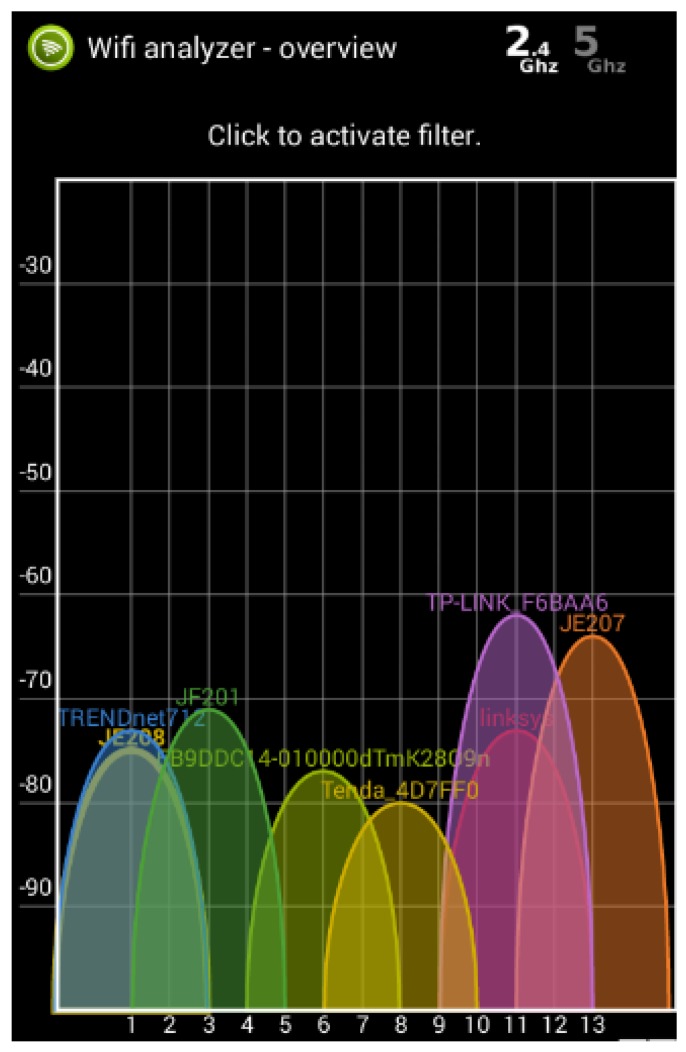
An example of evenly distributed Wi-Fi communications.

The environment was assessed using Wi-Fi Analyzer application on a smartphone (Figure 6). Typical environmental noise was considered as one with Wi-Fi communications evenly distributed on the specific channels, covering the whole 2.4 GHz spectrum. This seemed to be an appropriate simulation of the real road network, in which unknown communications may occur in every point, without any control (from the sensor network point of view) of the channels used. An example of this environment is presented in Figure 6 in which the channel numbers are represented on the horizontal axis and the signal strength, measured in dBm, on the vertical one.

The tests have been performed in the following scenarios:

- Typical, uncontrolled, environmental noise.
- Heavy traffic on Wi-Fi channels 1, 6 and respectively 11. The router was set to each channel, with a 40 MHz bandwidth, to consider the worst-case scenario that can occur. A 40 MHz wide channel will double the use of the available Wi-Fi spectrum, leaving fewer non-overlapping channels available for Bluetooth or ZigBee and worsening the interference problem.

**Figure 7:**
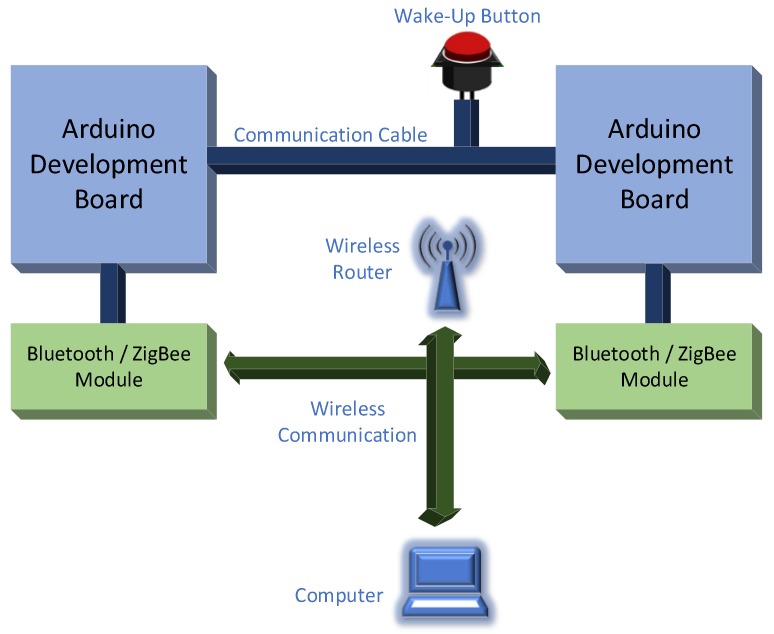
Handshake time measurement test bed.

The evaluation started with the test devices put to sleep. An additional wired connection between emitter and receiver was used to signal both modules to wake up. The connection was done as shown in Figure 7.

Two moments were considered, the difference between them representing the handshake time:

- The moment when the button is pressed, considered as reference. This will power up the modules that will connect with each other and the Master/Coordinator module will start transmitting time stamp messages to the Slave/End-device module.
- The moment when the first message is received and displayed by the Slave/End-device module.

Both communication modules require a wake-up time which should not be considered as a part of the handshake and needs to be subtracted from the obtained measurements. According to their specifications, the value is 504 µs for the Bluetooth module [16] and 13.2 ms for the ZigBee module [29].

Therefore, the handshake time will be:

(1) $$t_{h\_Bluetooth}=t_{Slave}-t_{Master}-504\;\mu s$$

(2) $$t_{h\_ZigBee}=t_{End-Device}-t_{Coordinator}-13.2\;\mathrm{ms}$$

**Figure 8:**
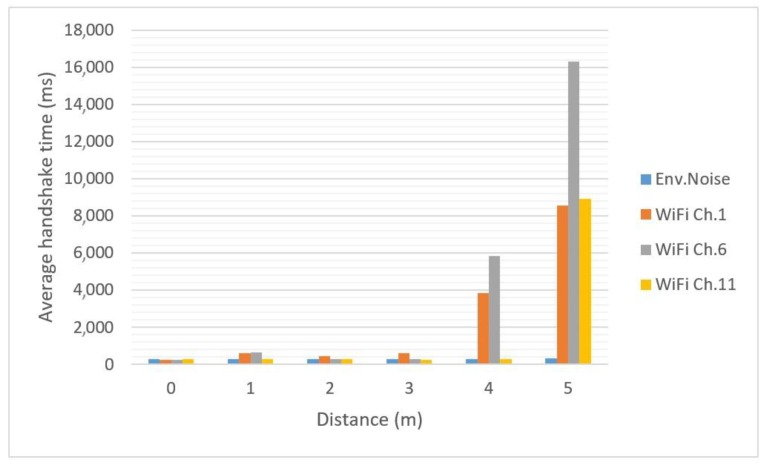
Bluetooth handshake time.

For the Bluetooth technology, handshake time was determined for each considered scenario, the distance between the modules being modified between 0 and 5 m, with 1-m step. Increasing the distance over 5 m led to an increase in handshake time that made this communication technology unsuitable for data exchange between moving devices, in the event of strong interference from the considered Wi-Fi channels. Average measurement values are presented in Table 1 and Figure 8.

With environmental interference present, which usually can be described as low Wi-Fi traffic, the handshake time has an average value of about 290 milliseconds. From Figure 8 it can be observed that handshake time is not significantly influenced by Wi-Fi traffic on any of the tested channels, if the distance between the modules is small, up to 3 m, the average value being around 380 milliseconds. For greater distances, Wi-Fi channel 6 has the highest influence over the handshake time, but the other channels will affect it too, determining average values which will make this technology difficult to implement if the considered applications will need a free communication environment at all times. The influence of specific Wi-Fi channels (1, 6, 11) occurs due to the initial connection for Bluetooth protocol, that used three advertising channels (presented in Figure 4) that are close to, or even overlap, Wi-Fi channels 1, 6, and 11, respectively. When heavy traffic is induced on these channels, the initial connection between Bluetooth devices is delayed. However, for communications disturbed only by environmental noise (as it would be likely the case in real life), the handshake time remains about the same for all tests.

Handshake time was determined for the same considered scenarios as for Bluetooth, with the exception that the modules were only placed at 0 m from each other. Few tests were made for distances up to 5 m and no significant differences between measurements were observed. Average measurement values are presented in Table 2. It can be seen in Table 2 that ZigBee channels 12, 17, 22 and 25, that overlap Wi-Fi channels (Figure 5), are easily influenced by Wi-Fi environmental interference. This behaviour is accentuated if heavy Wi-Fi traffic is generated.

From Table 2 it is obvious that ZigBee is also influenced by the nearest Wi-Fi channels: in normal operating conditions there are evenly distributed communications among Wi-Fi channels (considering that auto-configurable routers properly select non-congested channels to improve performance, resulting in a relatively even communication distribution among the whole spectrum). But in special interference conditions, when the routers were configured to induce the most influence, the values have significantly increased. The only exception is channel 26, which is the most protected, being the furthest one in the frequency spectrum, and which seem not to be influenced by Wi-Fi at all. Therefore, it is obvious that for relevant results, this is the one that should be used in future tests.

**Figure 9:**
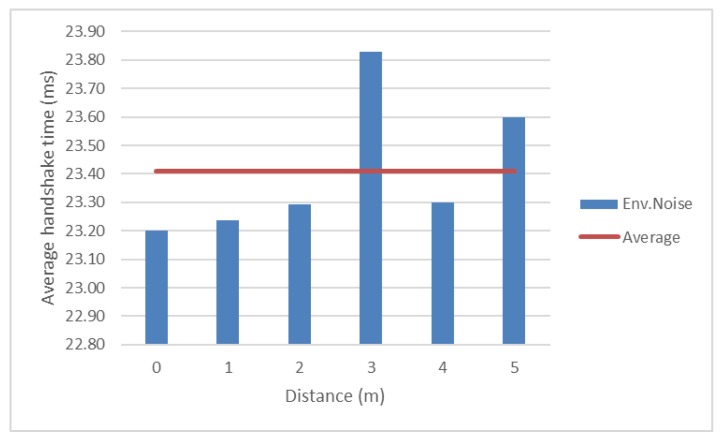
ZigBee handshake time.

To compare Bluetooth and ZigBee in the same conditions, handshake tests have been performed for ZigBee channel 25, in environmental noise. The behaviour is relatively constant, as the values varies between 23.20 ms and 23.83 ms. This is presented in Figure 9, where the red line represents the average value.

**Figure 10:**
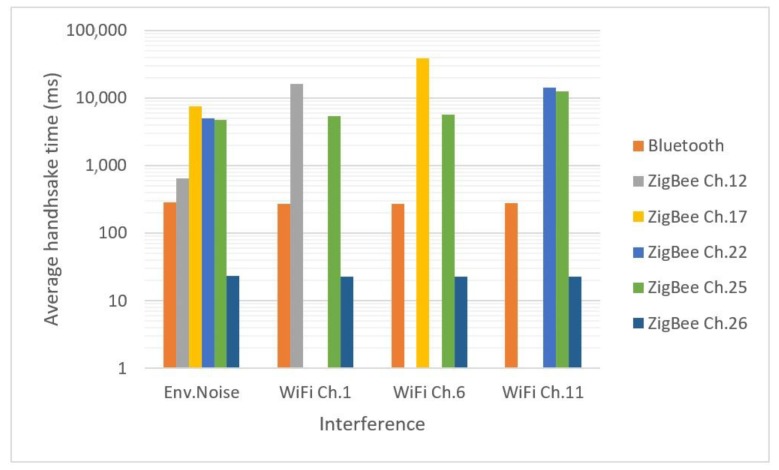
Bluetooth and ZigBee handshake time.

A comparison between Bluetooth and ZigBee handshake time measurements is presented in Figure 10. It can be seen that Bluetooth handshake is faster than the ZigBee’s one for all scenarios with one exception, ZigBee channel 26 which is furthest from Wi-Fi channels. For this channel, the handshake time is ten times smaller than Bluetooth lowest one, and it seems to be the right solution for implementing ZigBee communications.

### 3.4. Data Trasfer Time

Data transfer tests represented the second stage in technology evaluation. The connection time is important, especially when taking into consideration moving devices, due to short interval the emitter and receiver are in each other’s communication range. But after the devices are connected data transfer is the main parameter that can reveal the quantity of data that can be exchanged, with a fair probability of error-free communication. Data transfer tests were performed in two cases: static tests, that had the goal to determine the maximum communication distance, and dynamic tests, that intended to evaluate the capability of devices to exchange a certain amount of data when moving at different speeds against each-other.

#### 3.4.1. Static Tests

Static tests were performed in laboratory conditions, inside a building, in open space, with direct line of sight between the emitter and receiver modules. Similar to connection time assessment, the data transfer tests considered the following scenarios:

- Environmental background noise (communications spread across the 2.4 GHz spectrum).
- Heavy traffic on Wi-Fi channels 1, 6, and 11.

For each scenario, three situations have been addressed regarding the length of the message: 256, 512 and 1024 bits, considering the message length analysis from [10] from which it resulted that these values may be used in vehicle communications, being suitable for data exchange, depending on the application.

The evaluation took into consideration 100 consecutive message exchanges. For each of them, the emitter will send a message, of a specific length, and enter a listen mode. The receiver will evaluate the message and, if it will be correct, it will return the same message. If it will be wrong, it will send a different message back, to trigger a data resend. The emitter, being in listen mode, will wait for the response. If a resend will be necessary, it will send the initial message again. If the response will be correct, the time elapsed since sending the first message to this moment was calculated, as a two-way correct communication.

The tests have been performed with the modules already connected to each other. The main goal of this evaluation was to test the maximum distance for which the communication can still be performed. Although from previous data it seems to be irrelevant to test Bluetooth for distances greater that 5 m (as increased handshake time may cause impossibility for data transfer), there are some applications that does not require speed, instead the distance is key parameter (such as downloading data from busses at the end of the route). As the goal of this research was to test all possible conditions, we included these tests in the evaluation.

**Figure 11:**
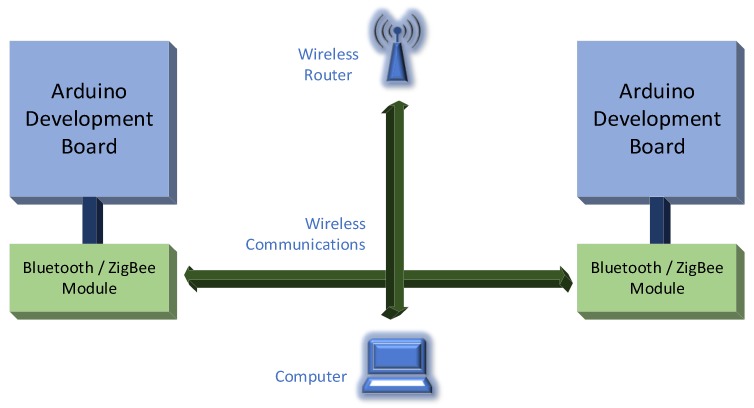
Static data transfer time measurement test bed.

The distance between the modules was modified between 0 and 15 m for the Bluetooth ones and between 0 and 50 m for the ZigBee ones, with 5-m step. Placing the Bluetooth modules at a distance greater than 15 m, and ZigBee modules at over 50 m from each other, has made it almost impossible to transfer messages. Test bed setup is presented in Figure 11.

As resulted from the handshake time tests, ZigBee channel 26 was used for the corresponding message transfer time measurement.

**Figure 12:**
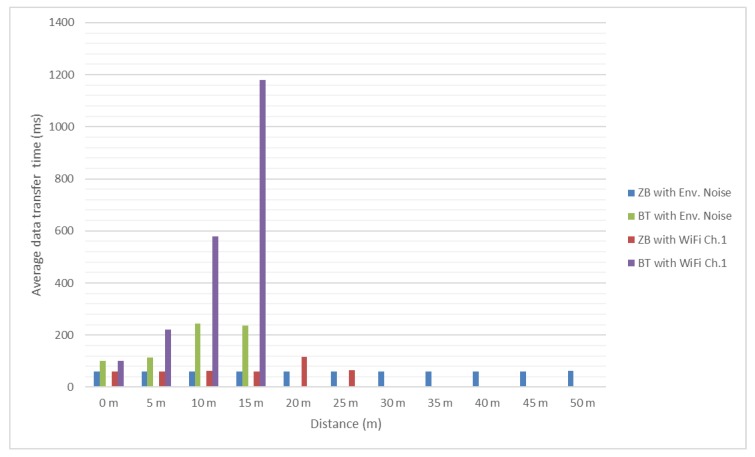
Bluetooth and ZigBee message transfer time (256 bits message/Environmental and Wi-Fi channel 1 interference).

**Figure 13:**
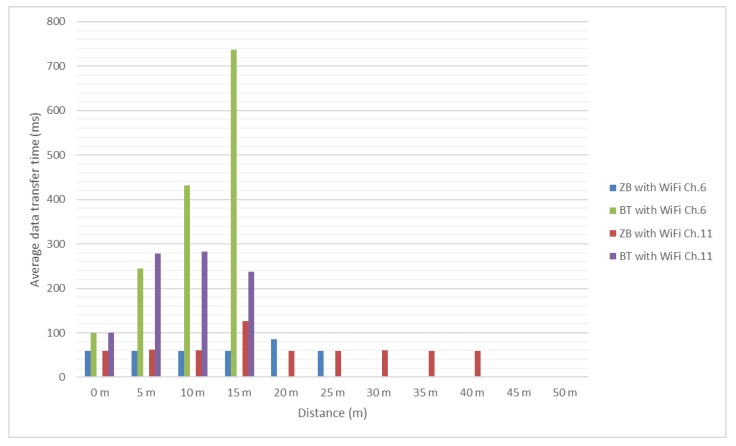
Bluetooth and ZigBee message transfer time (256 bits message/Wi-Fi channel 6 and 11 interference).

Test results are presented in the Figure 12 and Figure 13. It should be mentioned that, since no significant interference was determined between ZigBee channel 26 and considered Wi-Fi channels, the authors did not test these scenarios for distances greater than 25 m and use as reference the values obtained in presence of the environmental background noise.

In Figure 12 and Figure 13 it can be noticed that the message transfer time is relatively constant for ZigBee modules with values around 60 milliseconds (this was to be expected due to lack of overlapping between channels) and increasing with the distance for the Bluetooth ones to over 1 s, with channel 1 having the greatest influence. However, even considering this increase, Bluetooth allowed messages to be transferred successfully.

**Figure 14:**
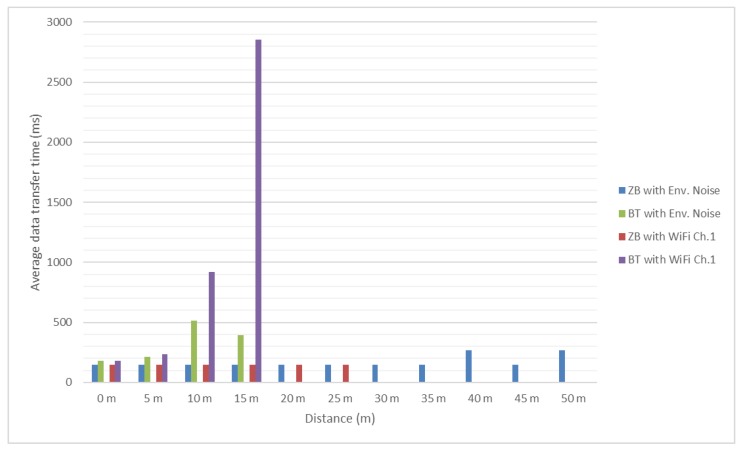
Bluetooth and ZigBee message transfer time (512 bits message/Environmental and Wi-Fi channel 1 interference).

**Figure 15:**
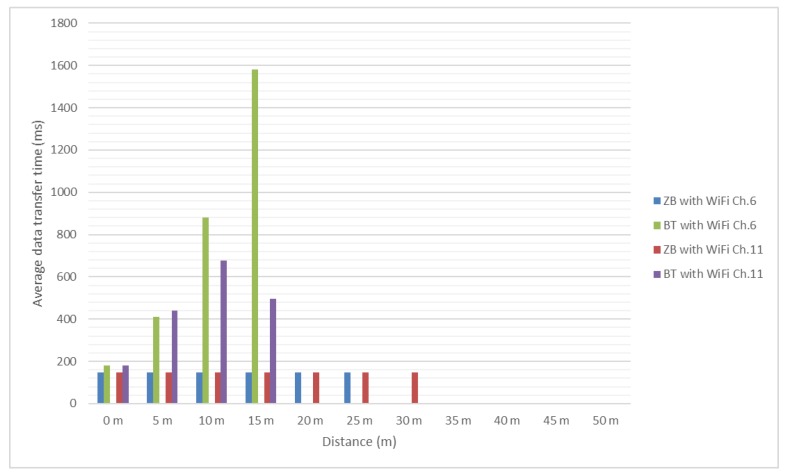
Bluetooth and ZigBee message transfer time (512 bits message/Wi-Fi channel 6 and 11 interference).

In Figure 14 and Figure 15 it can be noticed that the message transfer time for ZigBee modules continues to be relatively constant with values around 150 milliseconds. Bluetooth message transfer time has the same behavior as for previous tested message length, reaching values of almost 3 s. For Bluetooth, it becomes difficult to successfully transfer considered messages as the distance between modules is increasing.

**Figure 16:**
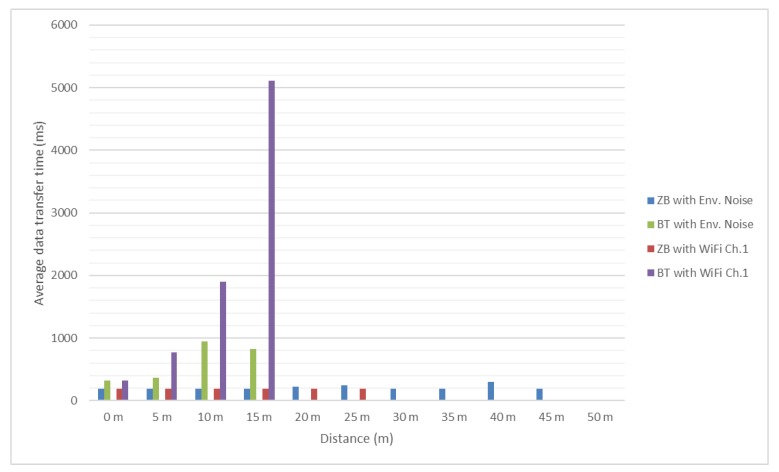
Bluetooth and ZigBee message transfer time (1024 bits message/Environmental and Wi-Fi channel 1 interference).

**Figure 17:**
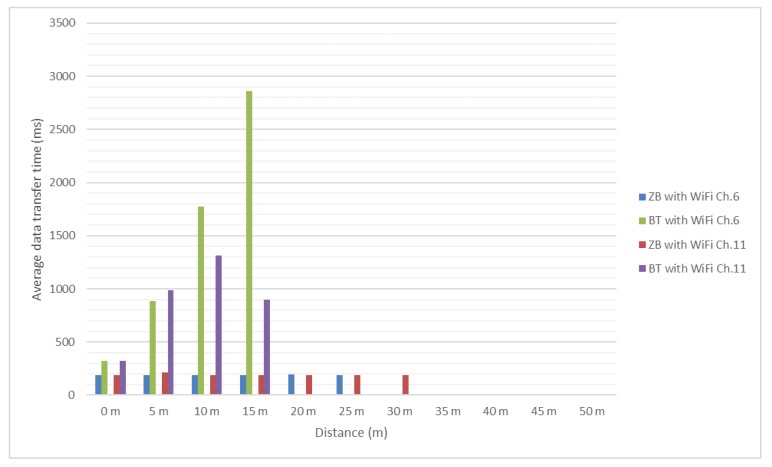
Bluetooth and ZigBee message transfer time (1024 bits message/Wi-Fi channel 6 and 11 interference).

In Figure 16 and Figure 17 it can be noticed that the message transfer time for ZigBee modules continues to be relatively constant with values around 190 milliseconds, but the maximum distance decreased to 45 m. Bluetooth message transfer time continues to have the same behavior as for previous tested message lengths, reaching values of more than 5 s. For distances greater than 10 m it is a very difficult task for Bluetooth to successfully deliver the message under heavy interference.

In conclusion, Bluetooth will allow for exchange of messages but only for low moving speeds or for vehicles that will spend short periods of time near the infrastructure equipment. ZigBee, on the other hand, will achieve successful communication at higher moving speeds, smaller messages being recommended and for non-critical applications.

#### 3.4.2. Dynamic Tests

The tests in motion were performed on a side road with very low traffic flows, in an industrial area, and with clear line of sight between the test modules. One of them was deployed in a fixed point and the other one was installed on a car. Urban environment was assessed, with car speeds between 10 and 50 km/h. For each evaluation the data exchange was tested with the vehicle coming in and out of the road side module communication range and the results were published by the authors in [30].

**Figure 18:**
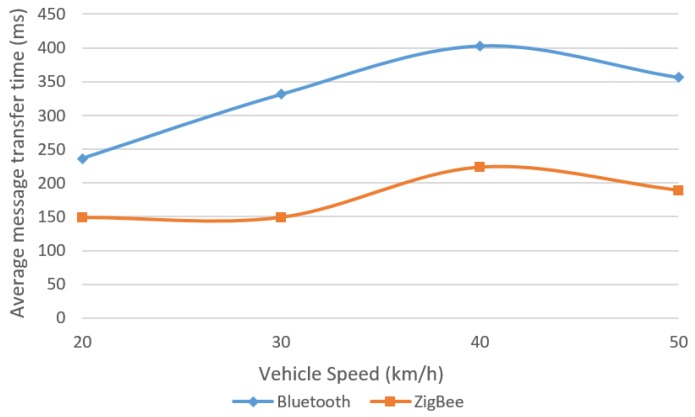
Average message transfer time for 256 bit messages [30].

**Figure 19:**
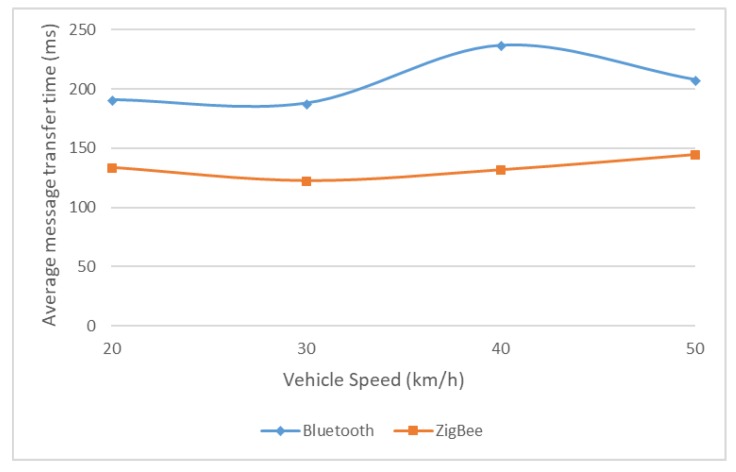
Average message transfer time for 128 bit messages source: [30], with extended data included.

The same ZigBee channel 26 was considered and different message lengths were tested. The use of 256 bit messages in this mobile environment led to acceptable message transfer times (Figure 18). Regarding greater length messages (512 and 1024 bits), as seen from previous results they led to difficulties in accomplishing a successful data transfer, especially for Bluetooth, and to increased and, in most of the cases, unsuitable message transfer time. Obviously, these problems worsened when the data transfer was attempted between two modules moving towards or away from each other, so these two types of messages were no longer considered because very few or none of them were successfully transmitted at first tests. At last, to increase the success rate of the message transfer, a smaller length was considered (128 bits message, Figure 19) keeping in mind that in many situation smaller amounts of data need to be transferred or it is feasible to split it into shorter messages.

As in previous tests, ZigBee provided more constant results, with average message transfer times below Bluetooth, and with a communication range significantly larger. The final conclusions are presented in the next section.

## 4. Conclusions

Handshake time measuring was the first step in communication evaluation. The capability of the devices to quickly connect to each other, starting from a sleep mode (that will increase energy efficiency) is essential in a dynamic environment like road traffic. From the tests performed it resulted that ZigBee channel 26 is undisturbed by other communications, while Bluetooth, with its frequency hopping approach, might encounter congested conditions, thus delaying the first step of the communication process. This result influenced the following test conditions, as from that moment, for ZigBee only channel 26 was used. Bluetooth, due to its specific protocol, cannot avoid interference by default. Static and dynamic data transfer tests have proved again that ZigBee has the following advantages against Bluetooth v4:

- Higher communication range
- Lower times for data transfer
- A more uniform set of values.

Considering the recent implementation of Bluetooth v5, the necessity to re-run these tests to properly evaluate the improvement of the new technology in similar conditions must be emphasized. All environmental conditions for the field tests were assessed using specific applications (as described earlier) to ensure the validity of these results. From all these we may conclude that ZigBee technology may provide a valuable support for large-scale energy efficient sensor networks. The next steps will be to evaluate critical messages transfer parameters to extend the possible usage of this technology in vehicular environment.

## Figures and Tables

**Table 1:** Bluetooth handshake time average values (milliseconds).

Distance (m)	Environmental Noise	Wi-Fi Channel 1 Interference	Wi-Fi Channel 6 Interference	Wi-Fi Channel 11 Interference
0	284.8856	268.8632	270.4752	279.6896
1	294.484	619.2584	657.5296	282.4408
2	280.2852	470.3724	277.1484	295.7724
3	277.894	628.2776	297.224	270.3096
4	284.582	3840.1712	5840.5988	277.8568
5	325.2228	8558.9332	16,323.9836	8938.8316

**Table 2:** ZigBee handshake time average values (milliseconds).

ZigBee Channel	Environmental Noise	Wi-Fi Channel 1 Interference	Wi-Fi Channel 6 Interference	Wi-Fi Channel 11 Interference
12	649.7	16,059.7		
17	7477.6		39,063.4	
22	5010.6			14,349.3
25	4721	5431.1	5630.4	12,565.7
26	23.2	22.8	22.6	22.7
